# Collecting mouse livers for transcriptome analysis of daily rhythms

**DOI:** 10.1016/j.xpro.2021.100539

**Published:** 2021-05-14

**Authors:** Thomas Mortimer, Patrick-Simon Welz, Salvador Aznar Benitah, Kevin B. Koronowski

**Affiliations:** 1 Institute for Research in Biomedicine (IRB Barcelona), The Barcelona Institute of Science and Technology (BIST), 08028 Barcelona, Spain; 2 Catalan Institution for Research and Advanced Studies (ICREA), 08010 Barcelona, Spain; 3 Hospital del Mar Medical Research Institute (IMIM), Cancer Research Program, 08003 Barcelona, Spain; 4 Center for Epigenetics and Metabolism, U1233 INSERM, Department of Biological Chemistry, University of California, Irvine, CA 92697, USA

**Keywords:** Cancer, RNA-seq, Metabolism

## Abstract

Molecular daily rhythms can be captured by precisely timed tissue harvests from groups of animals. This protocol will allow the investigator to identify transcriptional rhythms in the mouse liver while also providing a template for similar analyses in other whole metabolic organs. We describe steps for mouse entrainment, liver dissection, and rhythmicity analysis from total RNA sequencing data. The resulting rhythmic transcriptome will provide the user with a starting point for defining specific biological processes that undergo daily rhythms.

For complete details on the use and execution of this protocol, please refer to Koronowski et al. (2019). A similar protocol for interfollicular epidermal cells is demonstrated in Welz et al. (2019).

## Highlights

• Investigate a gene or pathway in the fourth dimension—time
• Use precisely timed tissue harvest to detect daily transcriptional rhythms
• Full description of mouse entrainment, liver dissection, and rhythmicity analysis
• A reference protocol for investigating other whole metabolic organs

## Graphical abstract

**Figure:**
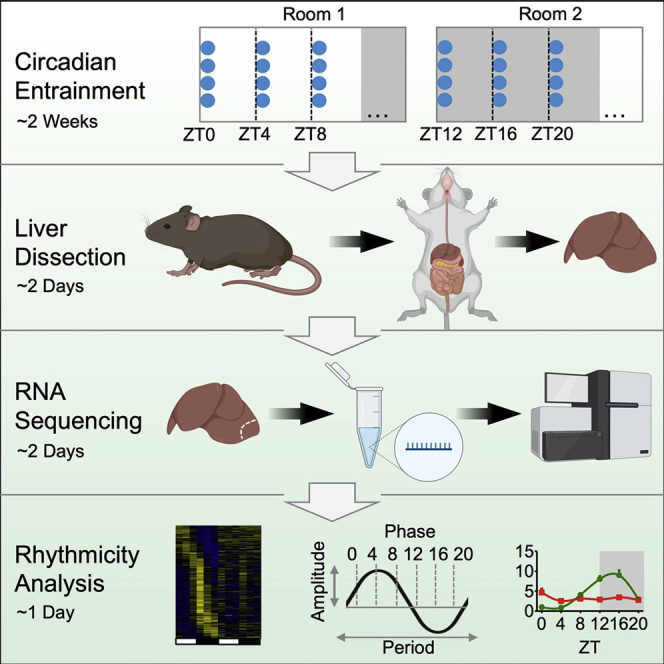

## Before you begin

**Figure 1:**
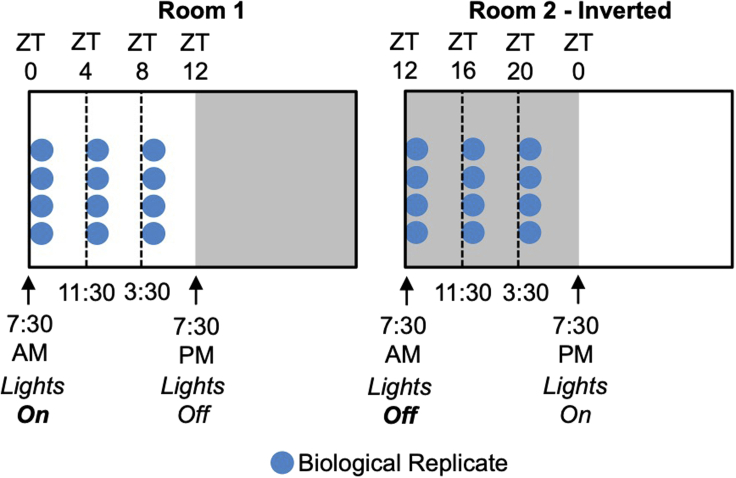
Scheme of entrainment to the light-dark cycle Livers are collected at six time-points during the light–dark cycle. One mouse is one biological replicate for one time-point. Note that the light schedule of Room 2 is inverted, with lights-off at 7:30 am, and lights-on 7:30 pm. Lights-on and -off times can be adjusted to whatever is convenient for the researcher. ZT, *Zeitgeber* time.

Familiarize yourself with circadian terminology. *Zeitgeber* time (ZT) denotes the time of day in a standard 12 hr light: 12 hr dark schedule (ZT0 = transition from dark to light periods; ZT12 = transition from light to dark periods) (Figure 1). This contrasts with the denotation of time under free-running conditions, such as constant darkness. In this case, time – referred to as circadian time (CT) – needs to be calculated based on the individual animal’s period and activity onset (Jud et al., 2005). Note that mice are nocturnal, meaning that they are active and feed during the night-time.

### Mouse entrainment

**Timing: 2 weeks**

1. House mice in individual cages in two rooms dedicated to the experiment, and avoid breeding/weaning or regular changing of cages in these rooms (Figure 1). In this minimal-disturbance environment, mice can develop robust behavioral rhythms (e.g., sleep–wake, feeding–fasting). If two rooms are not available, other strategies exist and are commonly employed for this type of experiment. In particular, mice can be housed in specialized cabinets or cages that allow the light schedule for each mouse to be programmed separately (see ‘troubleshooting’ for more detail). Additionally, the times presented here are those that we have found convenient. Equally, the light schedules can be adjusted so that dissection times are more suited to the investigator (e.g., 9:30 am/pm lights-on/off instead of 7:30).
  a. Room 1: set the light schedule to lights-on at 7:30 am, and lights-off at 7:30 pm.
    i. Place n = 4 mice in the room for day-time-points: ZT0 (7:30 am); ZT4 (11:30 am); and ZT8 (3:30 pm).
  b. Room 2: invert light schedule to lights-off at 7:30 am, and lights-on at 7:30 pm.
    i. Place n = 4 mice in the room for night-time-points: ZT12 (7:30 am); ZT16 (11:30 am); and ZT20 (3:30 pm).
  c. Set both rooms to a light intensity of ~100–400 lux, room temperature to 20°C–26°C (68°F–79°F), and (ideally) 30%–60% humidity.

***Note:*** These ranges of temperature and light intensity are standard for animal facility rooms. However, temperatures in this range are below the mousés thermo-neutral zone of 30°C and may result in cold stress and activation of brown fat metabolism (Ganeshan and Chawla, 2017); temperatures can be raised if this is an important aspect of your experiment.

***Note:*** If single-housing must be avoided, house mice in groups according to genotype or treatment type. Social cues and cohabitation are shown to impact the circadian clock in mice and hamsters (Paul et al., 2014, 2015). Thus, altered behaviour of a mutant mouse may influence that of wild-type littermates or vice versa.

***Note:*** Two entrainment rooms allow all time-points to be collected during reasonable working hours. However, the experiment is still achievable using one room, but will require experimenters to perform dissections during the night-time (e.g., 11:30 pm, 3:30 am).

***Note:*** Consider carefully the number of replicates collected per time-point (see ‘limitations’ for further information). Also take into account the number of replicates required for assays that you plan to carry out in parallel to RNA sequencing.

***Alternatives:*** It is important to distinguish daily or diurnal rhythms from circadian rhythms (see: ‘limitations’). This protocol, under light-dark conditions, is designed to identify daily rhythms. Circadian rhythms can only be identified under free-running conditions, such as constant darkness. This protocol can be adapted to identify purely circadian rhythms by maintaining the mice in constant darkness for two days prior to tissue harvest (Hughes et al., 2017).

***Optional:*** Feeding behaviour has a circadian rhythm in mice; ~75% of food is consumed during night-time, in the active phase (Atger et al., 2017). This feeding rhythm is a zeitgeber (time giver) or synchronizing cue for many metabolic tissues (Weger et al., 2021). To interpret results of this protocol, it is important to determine if your mutant mouse or experimental condition exhibits markedly altered feeding behaviour (as is the case in clock-disrupted animals) (Lamia et al., 2008). To eliminate food intake as a variable, restrict food access exclusively to the night-time for four days prior to tissue dissection (Atger et al., 2015).

troubleshooting 1

2. Allow mice to entrain (synchronize) to the light schedules for two weeks. Change cage bedding on day seven if necessary.

**CRITICAL:** Avoid entering rooms during the dark period if it exposes mice to light even briefly. Ensure any light-emitting devices in the entrainment rooms are covered. Even very low intensity light (~5 lux) can perturb certain metabolic rhythms (Borniger et al., 2014; Cissé et al., 2017). If necessary, dim red headlamps can be used to check on mice during the dark period. Night vision goggles equipped with an infrared beam are ideal, finances permitting (Jud et al., 2005).

**CRITICAL:** Avoid creating potentially stressful events for the mice, such as loud noises, strong odors (colognes and perfumes), or bedding changes close to the date of dissection (Sorge et al., 2014). Circadian rhythms are tied to stress physiology. For example, glucocorticoids are potent zeitgebers or synchronizing cues for circadian clocks (Dickmeis, 2009; Oster, 2020).

## Key resource table

Note that RNA extraction and sequencing reagents/protocols can be adapted as preferred.

**Table:** 

REAGENT or RESOURCE	SOURCE	IDENTIFIER
**Critical commercial assays**		

RNA library prep kit for total RNA sequencing	Illumina	RS-122-2001
RNA purification kit	QIAGEN	74136

**Deposited data**		

Koronowski et al., 2019, *Cell*	GEO	GSE117134

**Experimental models: organisms/strains**		

Mouse: C57Bl/6J background (10-week-old) male or female	Corresponding article	WT – Bmal1wt/wt, Alfp-cre−/tg

**Oligonucleotides**		

qPCR primers	This article	Table S1

**Software and algorithms**		

JTK_CYCLE	https://openwetware.org/wiki/HughesLab:JTK_CYCLE	Hughes et al., 2010
BIO_CYCLE	http://circadiomics.igb.uci.edu/biocycle	Agostinelli et al., 2016
MetaCycle	https://cran.r-project.org/web/packages/MetaCycle/index.html	Wu et al., 2016
R	https://cran.r-project.org/	R Project
DryR	(https://github.com/naef-lab/dryR)	Weger et al., 2021

**Other**		

Aluminum foil	Any	N/A
Dissection tools (forceps, small scissors, big scissors)	Any	N/A
Red-light headlamp	Any	N/A
Black trash bag/blackout curtain (for mouse transport)	Any	N/A
Autoclaved 2 mL tubes	Any	N/A

## Step-by-step method details

### Liver dissection

**Timing: 2 days**

Liver dissection occurs over two days: on day one, collect livers from the three time-points in Room 1 (R1), and on day two, collect livers from the three time-points in Room 2 (R2). Alternatively, all samples can be collected in one day if the light schedules of the two rooms are staggered by 1 hr (e.g., R1: lights-on at 7:00 am, lights-off at 7:00 pm; R2: lights-off at 8:00 am, lights-on at 8:00 pm). This allows enough time to collect samples back-to-back for R1 ZT0 and R2 ZT12, R1 ZT4 and R2 ZT16, and R1 ZT8 and R2 ZT20. See Note below.

**Figure 2:**
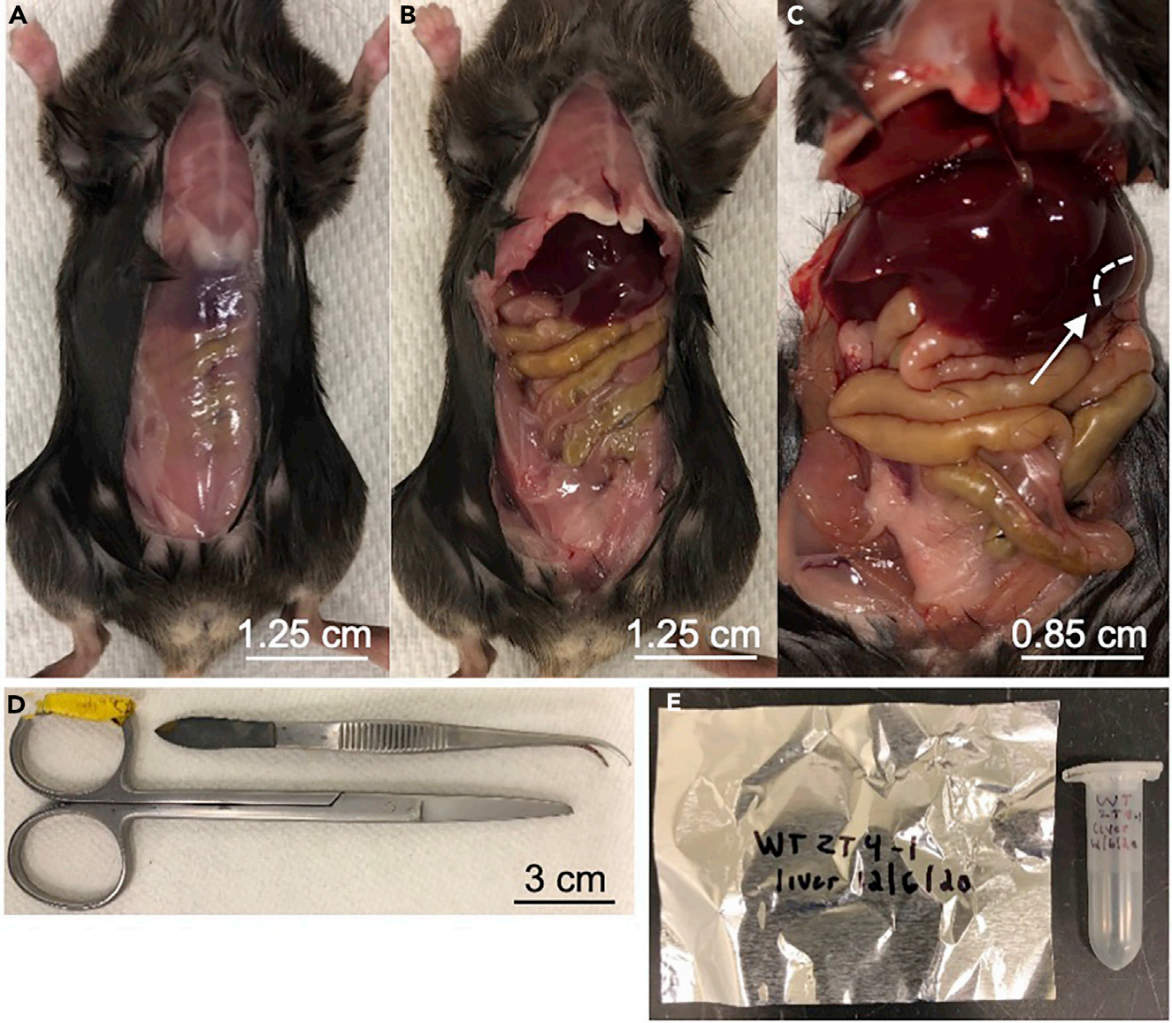
Steps of liver dissection (A) Midline incision of the skin. (B) Midline incision of the muscle to expose organs. (C) Ribcage cut on each side of the mouse. The white arrow and outline indicate the liver piece to be placed in the 2 mL tube. Alternatively, the liver piece can be taken from any desired lobe, or whole liver can be homogenized after freezing. (D) Any generic dissection tools are suitable. (E) Left, foil for freezing the rest of the liver for other assays. Right, 2-mL tube containing small liver piece for RNA extraction and sequencing.

1. Take mice cages one at a time to a dedicated dissection/collection room (Figure 2).
  a. Euthanize mouse by CO_2_ asphyxiation, cervical dislocation, or another approved method. For dark-phase time-points, euthanize mice in darkness.
    **Timing: 60 s**
  b. Decapitate mouse as a secondary confirmation. This is an important step when collecting dark-phase time-points.
    **Timing: 10 s**
  c. With the mouse on its back, take small scissors and remove the layer of skin up the midline (Figure 2A).
    **Timing: 30 s**
  d. Make a second cut of the muscle to expose the abdominal cavity and organs (Figure 2B).
    **Timing: 30 s**
  e. Cut down both sides, at the ribcage, to gain access to the entire liver (Figure 2C).
  f. Identify the different lobes of the liver. While gently holding the left lateral lobe with forceps, cut a small piece of liver (~0.5 cm, enough for RNA extraction) (Figure 2C).
    **Timing: 1 min**
  g. Place liver piece in a 2 mL tube and immediately snap-freeze in liquid nitrogen (Figure 2E).
    **Timing: 30 s**
  h. Remove the remainder of the liver, taking care to avoid any non-liver tissue and connections. For example, while taking care to not spill its contents, remove the gallbladder and its connections with the liver. Wrap the liver in aluminum foil (Figure 2E). Immediately snap-freeze foil in liquid nitrogen.
    **Timing: 1 min**
  i. Store samples at –80°C.

**Pause point:** Livers can be stored long-term at –80°C.

**CRITICAL:** When collecting dark-phase time-points, do not expose mice to light at any point. While transporting mice to the dissection room, they can be covered with heavy black trash bags or a blackout curtain. Do not turn on dissection room lights until mice have been decapitated. If collecting brain, remove the eyes before turning on the lights.

**CRITICAL:** Ensure collection is complete within ±30 min of the time-point. Consider the number of mice, data gathering (e.g., blood glucose, body weight), and tissue dissection time. For instance, if there are 10 mice that each take 6-min to process, start the harvest at 30 min prior to the time-point in order to finish at 30 min post-time-point. If possible, share the work between several investigators to reduce the amount of time spent per mouse.

***Optional:*** In addition to liver, collect other tissues of interest. Be sure to factor in extra dissection time. Consider beforehand the downstream assays for those tissues and apply appropriate collection methods.

### RNA extraction and library preparation for total RNA sequencing (RNA-seq)

**Timing: 2 days**

2. Extract RNA from the piece of liver in the 2 mL tube using an RNeasy plus mini kit (Qiagen), in accordance with the manufactureŕs instructions (https://www.qiagen.com/us/shop/automated-solutions/sequencers/rneasy-plus-mini-kit/)

**Figure 3:**
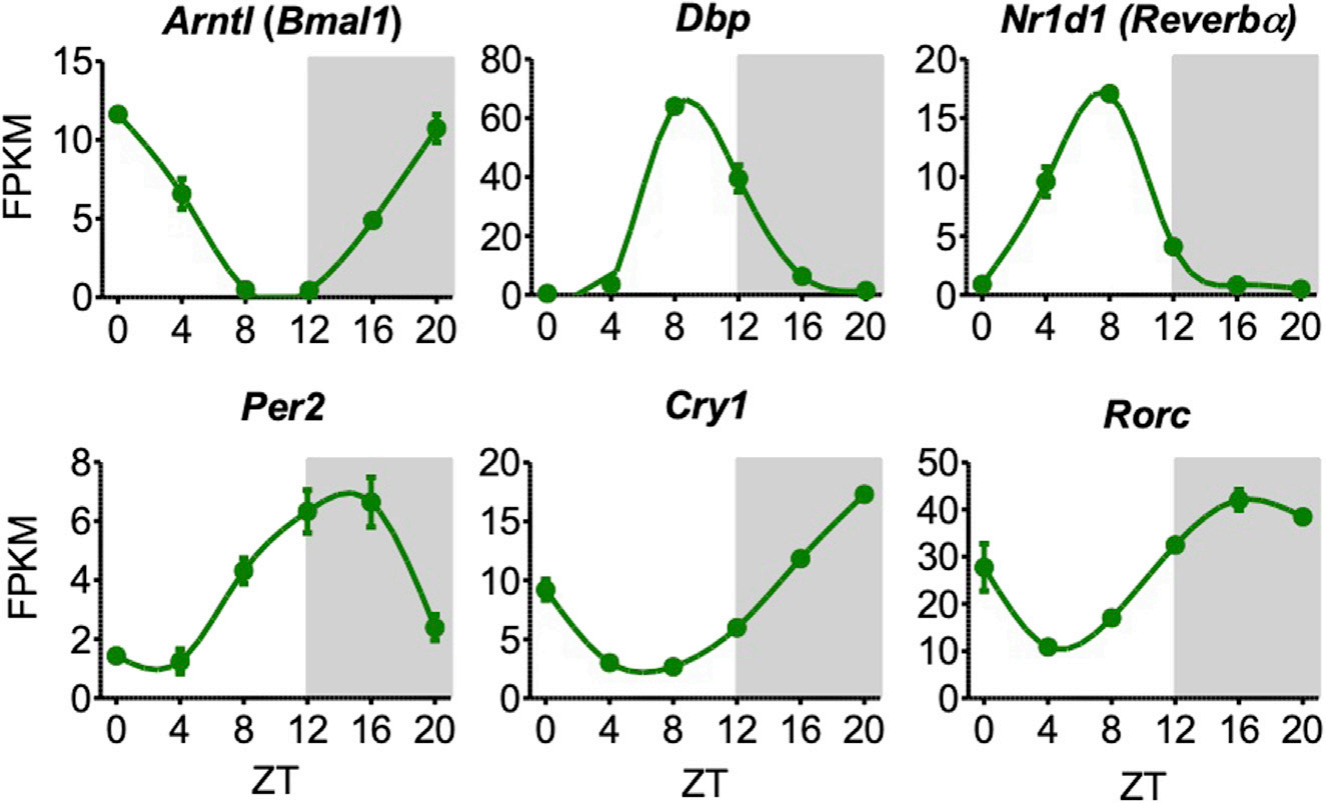
Clock gene expression from RNA sequencing Examples of clock gene expression generated from this protocol, showing high amplitude rhythms with particular peak phases (n=3). In our hands, *Dbp* is a good indicator of robust circadian entrainment, with a daily fold induction of >100–300×. The other clock genes shown, including *Per2*, have lower amplitudes and are also good indicators.

**CRITICAL:** Before proceeding to sequencing, validity of the collection should be checked by qPCR for clock genes (e.g., *Bmal1*, *Dbp*, *Cry1*, *Per2, Rorc, Reverbα*). High-amplitude rhythms with distinct phases should be apparent in *wild-type* mice (Figure 3; Table S1).

troubleshooting 2

3. Prepare libraries for sequencing, in accordance with the manufactureŕs instructions of a total RNA library preparation kit (https://www.illumina.com/products/by-type/sequencing-kits/library-prep-kits/truseq-rna-v2.html), and subsequently sequence the libraries. Sufficient depth is ~20 million reads/sample for single-end sequencing.

***Note:*** The type of sequencing, and library choice, should be directed by the hypothesis the researcher intends to test. Both paired-end sequencing and libraries enriched for specific RNA types (e.g., polyA-enriched) are compatible with this protocol.

4. Process the resulting sequencing data through a standard RNA-seq pipeline to quantify gene expression. FPKM, RPKM, and/or TPM values are compatible with downstream rhythmicity analysis.

**CRITICAL:** High-quality, correctly processed RNA-seq data is indispensable for identification of rhythmic transcripts using this protocol. Users are referred to bioinformatics-core-shared-training.github.io/RNAseq-R/ and bioconductor.org/packages/devel/workflows/vignettes/rnaseqGene/inst/doc/rnaseqGene.html as references for standard good practice in quality control and processing of RNA-seq data.

***Note:*** Apply Principal Component Analysis, Hierarchical Clustering or more specialised tests (see: Chen et al., 2020; George et al., 2015) to the RNA-seq data, to identify outliers that could potentially compromise rhythmicity analyses.

## Expected outcomes

Success in this protocol will yield high-amplitude rhythms of clock gene expression with distinct phases (Figure 3), as well as rhythms in hundreds to thousands of other transcripts (Koronowski et al., 2019). As mentioned above, this protocol is designed to identify daily rhythms (see: ‘limitations’). The circadian clock drives many of these rhythms, however this protocol cannot distinguish precisely the drivers of each transcript. Some transcript rhythms are not resulting from direct clock-output genes, but may be driven by other factors such as the light-dark cycle (Li et al., 2020; Thaben and Westermark, 2016) or feeding-fasting cycle (Greenwell et al., 2019; Vollmers et al., 2009; Weger et al., 2021). Moreover, recent studies suggest that a number of transcripts can oscillate independently of the canonical core clock machinery in *ex vivo* and *in vitro* systems (Ray et al., 2020). Thus, indirect and possibly core-clock-independent daily transcripts may also be identified by this protocol. Additional experiments need to be performed to determine if a particular rhythm is circadian (constant darkness) or a direct clock output gene (disrupted in tissue-specific clock mutant mice).

## Quantification and statistical analysis

### Daily rhythmicity analysis

**Timing: 1 day**

Here we provide steps to identify transcripts with daily rhythmicity in whole transcriptome data, using the R package JTK_CYCLE (Hughes et al., 2010). Although basic knowledge of the R programming language is required, running JTK_CYCLE should not pose an obstacle even to first-time users. Many excellent R tutorials for beginners are available for free, including through The R Project: https://cran.r-project.org/manuals.html. Often, researchers will want to perform differential rhythmicity analyses to identify how the rhythmic transcriptome changes after genetic, environmental or pharmacological interventions. A discussion of how this can be achieved can be found in the ‘limitations’ section of the protocol.

1. Install the R programming environment.
  a. R can be downloaded at: https://cran.r-project.org/
2. Download the scripts required to run JTK_CYCLE and move them to an appropriately named directory.
  a. The folder containing these scripts and a detailed user guide can be found at: https://openwetware.org/wiki/File:JTKversion3.zip

**Recommended:** The manuscript and user guide written for JTK_CYCLE by its creators describe in detail the underlying mathematics and give thorough technical advice, respectively. We highly recommended reading both of these informative publications before proceeding.

**Figure 4:**
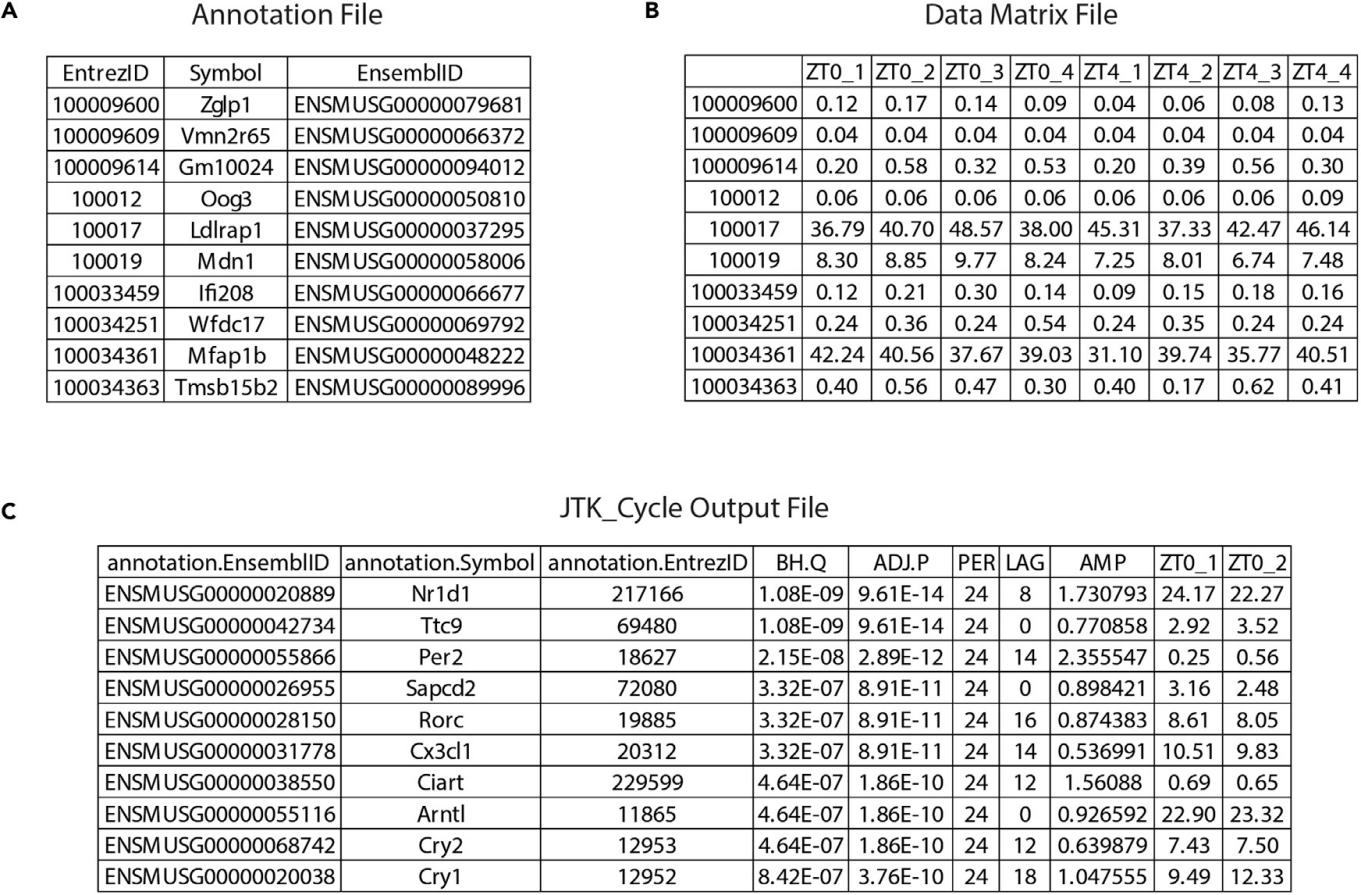
Input and output files of JTK_CYCLE (A) Correctly structured "annotation file", as required to run JTK_CYCLE. Only the first 10 rows of data are shown, along with the appropriate column headers. In this example, EntrezID, HUGO symbol (Symbol), and Ensembl ID gene annotations are included. (B) A correctly structured "data matrix file", as required to run JTK_CYCLE. Only the first 10 rows and 9 columns of data are shown, along with the appropriate column headers. The first column lists the EntrezIDs of all genes with expression quantified by RNA-seq. Subsequent columns indicate the RPKM expression values of each gene at each time-point, in chronological order (ZT0, ZT4, ZT8…). Replicates are placed in adjacent columns (ZT0_1, ZT0_2…). (C) A "JTK_CYCLE Output File" generated from the example input files in (A) and (B), and sorted by the statistical significance of circadian gene expression. The first three columns list the different gene annotations present in the "annotation file", and the following five columns give the circadian parameters of each gene as calculated by JTK_CYCLE. In the final columns, the RPKM values used by the algorithm are repeated. The first 10 rows and 10 columns of data are shown, along with the column headers. In this example, JTK_CYCLE identified 2035 rhythmic transcripts (ADJ.P <0.01) in WT liver, which represented 11.25% of the total measured transcripts. BH.Q, Benjamini–Hochberg q-value; ADJ.P, adjusted p-value; PER, period; LAG, phase; AMP, amplitude.

3. Using the previously-generated circadian RNA-seq data, prepare the annotation and data matrix files that will serve as the input to JTK_CYCLE (Figures 4A and 4B).
  a. The annotation file should comprise a first column listing the IDs of all genes quantified by RNA-seq, followed by columns containing other gene annotations (HUGO symbol, Ensembl ID, etc.) that the user prefers (Figure 4A).
  b. The data matrix file should comprise a first column listing the IDs of all genes quantified by RNA-seq (identical to the first column of the data matrix file), followed by columns chronologically (ZT0, ZT4, ZT8…) listing their respective expression values for all replicates at all time-points. Replicates must be placed in adjacent columns (Figure 4B).

**CRITICAL:** Ensure that the order of gene IDs is identical in the first columns of the data matrix and the annotation files.

4. In the same folder containing the scripts required to run JTK_CYCLE, save both the data matrix and the annotation files as tab-delimited text files (.txt).
5. In a text editor, open the “Run_JTK_CYCLE (Example1).R” script file.
  a. This file can be found inside the folder downloaded from the JTK_CYCLE webpage.

6. Modify the following terms in the script to reflect the dataset being provided to the algorithm:
  a. On line four, alter the project name to an appropriate description (default: "Example1").
  b. On lines seven and eight, change the file names to those of the annotation and the data matrix files, respectively (default: "Example1_annot.txt" and "Example1_data.txt").
  c. For this example, an experiment with six time-points collected over 24 hr and four replicates per time-point, the following lines of the script must also be altered to reflect the dataset structure:
    i. Line 12, "jtkdist(6,4)" indicates the number of time-points (six) and the number of replicates provided for each time-point (four).
      ***Note:*** If group sizes are unequal due to excluded or missing replicates, an additional line of code must be added to specify the number of replicates at each time-point. For example, if only three replicates are available for the first time-point, the line "group.size <- c(3,4,4,4,4,4)" would be added, and the term "jtkdist" changed to "jtkdist(length(group.sizes),group.sizes)".
    ii. Line 14, "periods <- 6:6" specifies the range of circadian periods that are of interest. In this instance, only genes with a 24-hr period are of interest; thus, "6:6" is the input (six time-points per cycle).
    iii. Line 15, "jtk.init(periods,4)" indicates the separation in hours (four) between time-points.

7. Change the working directory of R to the directory containing the data matrix file, annotation file, and scripts required to run JTK_CYCLE.
8. Paste the modified code into the R terminal and run the script.
  a. JTK_CYCLE will output a tab-delimited text file containing the results into the same directory (Figure 4C). This file provides the calculated Benjamini–Hochberg q-value (BH.Q), adjusted p-value (ADJ.P), period (PER), phase (LAG), and amplitude (AMP) for all genes analyzed.

**CRITICAL:** The JTK_CYCLE script must be modified correctly to accurately reflect the structure of the RNA-seq data generated.

***Note:*** JTK_CYCLE will typically take 10-15 min to run using a standard desktop computer or laptop.

9. To obtain a final list of robustly-rhythmic genes, the output file must be filtered to retain only those genes with clear daily rhythms in expression. Typically, an adjusted p-value of 0.01 provides a sufficiently stringent cut-off that retains only genes with clear, biologically-relevant rhythmic expression. In addition, selecting genes by expression amplitude can complement a p-value filter, adding an additional level of stringency to the rhythmic transcriptome identified.

***Note:*** The high stringency of JTK often leads to large numbers of false negatives if the computed q-value is used for filtering (Hutchison et al., 2015). In our experience, the adjusted p-value calculated by JTK serves as a more appropriate filter for identifying genes with reproducible rhythmic expression.

**CRITICAL:** The p-value filter selected must be directed by the level of noise intrinsic to the dataset obtained, which will vary on a case-by-case basis. Visual inspection of core clock genes, such as *Bmal1*, *Per2* and *Cry2*, can provide a good starting point to assess the technical and biological noise present in a circadian dataset (Figure 3).

10. The final set of circadian genes can now be used for further downstream bioinformatics analyses, such as gene ontology and pathway analysis, or to direct future *in vivo* and *in vitro* experiments.

***Note:*** For examples of phase plots, amplitude plots, and expression heat maps typically used to visualize circadian gene sets, please refer to Welz et al., 2019 and Koronowski et al., 2019 (Koronowski et al., 2019; Welz et al., 2019).

## Limitations

Here we describe how to collect liver tissue over one circadian cycle, with a large number of replicates per time-point and a relatively long sampling interval (4 hr). The experimental design used to characterize the daily rhythmic transcriptome varies extensively across published circadian studies. As such, the design employed to answer each new research question should be determined by balancing cost versus time considerations, and considering the particular focus of the study. An alternative, and arguably more stringent, strategy to that employed in this protocol would be to collect samples over two consecutive circadian cycles (48 hr) and to reduce the sampling interval (e.g., collect every 3 hr). This would make the data more robust to outliers and reduce false-negatives in rhythm detection (Hughes et al., 2017). However, due to cost and time limitations, the number of biological replicates is often sacrificed in such studies. This often makes it more difficult to calculate circadian parameters that require large numbers of biological replicates to provide the appropriate statistical power and precision. A detailed set of established guidelines for circadian ‘omics’ experiments is provided by Hughes et al., 2017, and it is highly recommended that the user study these guidelines in some detail before deciding on their final experimental design (Hughes et al., 2017).

The protocol described here requires liver sampling from different mice at multiple time-points. Currently, it is not possible to perform repeated measurements of the liver transcriptome in the same mouse over the circadian cycle. Thus, this experimental strategy has its limitations when obtaining samples and interpreting data from mice that may lack phase-alignment, or if the amplitude of the circadian rhythms in the individual experimental animals varies (e.g., after prolonged free running conditions in complete darkness). Under such experimental conditions, alternative methods might have to be applied to determine circadian rhythmicity. For example, clock gene rhythms can be visualized by sequential ear snip biopsies from the same mouse over a circadian cycle (Welz et al., 2019).

In this protocol we have described a procedure for identifying the daily rhythmic transcriptome under physiological light-dark conditions. To formally demonstrate that the rhythmic transcription identified is indeed driven by the circadian clock, or oscillates in a circadian manner independently of the core clock, it must meet a number of established criteria (Dunlap, 1999). First, the free-running period of the oscillation must be temperature compensated i.e., remains ~24 h across a broad range of physiological temperatures. Second, the oscillation must be ‘entrainable’ to external cues i.e., the phase of the gene shifts in response to changing environmental conditions, such as light cycles. Finally, a circadian oscillator must be self-sustained i.e., continuing to oscillate under constant environmental conditions, such as continuous darkness. A full description of the established methods used to test these conditions is outside the scope of this protocol, but users are referred to Eckel-Mahan and Sassone-Corsi as a reference for such approaches (Eckel-Mahan and Sassone-Corsi, 2015).

A wealth of algorithms has been developed to identify circadian features of "omics" data, each of which has specific intentions, strengths, and weaknesses. Although JTK_CYCLE is one of the most established and frequently cited examples for rhythmicity detection, we recommend validating results using different algorithms (Guan et al., 2020; Koronowski et al., 2019; Welz et al., 2019). Specifically, users are directed to BIO_CYCLE and MetaCycle as complementary approaches for rhythmicity detection (Agostinelli et al., 2016; Wu et al., 2016). BIO_CYCLE applies machine learning techniques, operates as a web tool, and provides a rapid and effective alternative to JTK. MetaCycle, in contrast, is an R package that enables the user to apply three popular rhythmicity detection algorithms to the same data set (ARSER, JTK_Cycle and Lomb-Scargle), allowing for independent confirmation of rhythmicity and avoidance of false-negatives. Moreover, when comparing the rhythmic transcriptomes of multiple groups (genotypes or conditions) users should consider employing algorithms specifically designed for differential rhythmicity analysis. The R package DryR (Differential Rhythmicity Analysis in R) enables a combined rhythmicity detection and differential rhythmicity analysis, allowing the user to detect changes in rhythmicity between multiple groups (Weger et al., 2021).

## Troubleshooting

### Problem 1

Dedicated investigator rooms are not available (step 1 in “before you begin”).

### Potential solution

Holding/breeding rooms can be used for entrainment. However, disruptions from staff or other researchers may compromise circadian behavior and thus will present a major limitation to the experiment. Alternatively, a range of cage and cabinet solutions have been developed to overcome such space limitations (cage - www.tecniplast.it/uk/product/leddy-lighting-control-system-with-in-gm500-red-cage.html, cabinet - www.actimetrics.com/products/circadian-cabinets/). These cages and cabinets enable the investigator to subject mice to alternative light schedules without the need for specific rooms, whilst also minimizing unwanted disruption to the mouse’s normal daily rhythms.

### Problem 2

Clock gene expression is non-rhythmic or irregular (step 2 in “step-by-step methods details”).

### Potential solution

Check that entrainment parameters are within the desired range. Measure humidity and temperature, and confirm that the room lights are turned on and off at the correct times using light meters able to log patterns over multiple days. Monitor entrainment rooms for disturbances during the day (for instance, other laboratory members spending time in the rooms, or entering and exiting the rooms frequently). Notify colleagues of entrainment weeks; hang signs on doors to indicate that there is an ongoing, highly sensitive experiment. During entrainment, regularly ask the facilities management about any major disruptions in power that might have occurred.

### Problem 3

If not enough age- and sex-matched mice can be generated at once (e.g., when using mice with a genotype of low mendelian ratio), sample collection over two days, as described in this protocol, might be impossible (step 1 in “before you begin”).

### Potential solution

When breeding mutant mice, it may not be possible to collect all samples over two days as described in this protocol. As long as experimental conditions can be kept constant, tissues can be collected over a period of months, and in our hands, this is suitable to detect rhythms by RNA sequencing. This approach may not be appropriate for other applications, such as measurement of certain unstable metabolites. When collecting over months, randomize genotypes and time-points to limit batch effects, and collect livers continually as mice become available (e.g., when reaching the right age).

### Problem 4

JTK_Cycle calculates few or no genes to have daily rhythms of transcription (step 9 in “quantification and statistical analysis”).

### Potential solution

Typically, an investigator should expect to detect 1000+ genes oscillating in a daily manner when this protocol is applied to the livers of *wild-type* mice. Nonetheless, this number will vary substantially depending on the whether an animal has been genetically modified or subjected to some environmental/pharmacological perturbation (see: Welz et al., 2019 and Koronowski et al., 2019 for examples of the scale of this variation upon genetic modification). Aside from true variation in the size of the rhythmic transcriptome, the degree of biological and technical variation present in the RNA-seq data gathered will determine the sensitivity with which daily rhythms can be identified by JTK_Cycle. Thus, it is essential that investigators: 1) Use a number of biological replicates that reflects the anticipated biological variability between mice, 2) Perform stringent quality control of the RNA-seq data input to JTK_Cycle, 3) Adjust the stringency of the filters used to classify daily rhythms according to the variability present in the data.

### Problem 5

JTK_Cycle fails to execute correctly, and the expected output files are not generated (steps 8 and 9 in “quantification and statistical analysis”).

### Potential solution

Ensure that the input files have been correctly formatted in line with the examples provided in Figure 4. In addition, check that the modifications required to the JTK_Cycle script have been made correctly, without syntax errors, and that the modifications correctly reflect the structure of the RNA-seq data. If the problem persists, users should consult the user manual of JTK_Cycle or contact its developers directly.

## Resource availability

### Lead contact

Requests for further information and/or resources and reagents should be directed to Kevin Koronowski (Kkoronow@uci.edu).

### Materials availability

This study did not generate new unique reagents.

### Data and code availability

Datasets associated with this protocol are available as supplemental tables in Koronowski et al., 2019 and the accession number for the sequencing data is GEO: GSE117134 (Koronowski et al., 2019). Please also see ‘key resources table’ for analysis algorithm information.

## Supplemental information

Table S1. Clock gene primers Primers to measure expression of clock genes by qPCR. Related to step 2 in “step-by-step methods details”.
